# General elections in Poland (1990–2024) in the unified territorial aggregation: GeoElections Poland 1.0

**DOI:** 10.1038/s41597-025-06418-2

**Published:** 2025-12-23

**Authors:** Przemysław Śleszyński, Mariusz Kowalski

**Affiliations:** Polish Academy of Sciences, Institute of Geography and Spatial Organization, Twarda 51/55, 00-818 Warsaw, Poland

**Keywords:** Geography, Politics

## Abstract

We share dataset of all general elections held in Poland between 1990 and 2024. These datasets correspond to two degrees of spatial aggregation: 41 constituencies for the Sejm and 94 for the Senate. In this dataset, for the first time, it was possible to collate voting results for electoral committees in unified territorial units. Such a compilation is unique and difficult to compile due to the multiple changes of administrative boundaries in Poland. In this dataset, we also organize more than 300 unique election committees into a uniform division into 5 basic political options. This data is publicly available but the way they are presented makes it impossible to create maps and comparisons, subject to time-consuming processing into any of the GIS systems. Therefore, we attach to the data a vector (SHP) map of electoral districts for the both chambers of the Polish parliament, to which it is easy to attach the developed relational dataset in XLS format in any year and aggregation.

## Background & Summary

Data presenting election results spatially, including at low levels of territorial disaggregation are the basis not only for scientific analysis, but also for civic control of elections, satisfying curiosity about the spatial variation of voting behaviour by the public. In terms of scientific research, they are the source basis for electoral geography. Based on detailed data on turnout, support and voting results, it is possible to analyze the broad determinants and factors of the public’s voting attitudes^1^. Access to election results from longer periods of time makes it possible to compare them and study the changes taking place^2^. This provides an opportunity to detect the persistence of electoral behaviour, emerging variability, as well as possible anomalies revealed only against a broader temporal and spatial background^3^. Comparison with other variables makes it possible to see correlations (or lack thereof) and to make hypotheses of a causal nature regarding the phenomena seen^4^. There are examples of the development of such datasets and other similar solutions for various countries and regions, among others in the United Kingdom^5^, the United States^6,7^, Sweden^8^, the Czech Republic^9^ or Germany^10,11^. The latter are probably the most detailed longitudinal and harmonized geospatial databases in the world, as they contain information on the results of all municipal, state, and federal elections in Germany since World War II, presented in a unified dataset of approximately 10,800 contemporary municipalities (Gemeinde). There are also collective datasets for many countries around the world, including the largest one currently, the Global Elections Database^12^, which contains data on election results from 36 countries, generally after World War II, and allows these results to be visualized down to the level of first-order administrative units, such as 51 states/districts in the US or 20 regions in Italy. For Poland, data is available for parliamentary elections from only 2001, 2005 and 2007, broken down into 17 NUTS1 statistical units (these are approximately equivalent to Polish provinces).

The existence of a dataset containing the results of successive elections that can be compared is important for all democracies and has significant cognitive significance. Thus, it is especially important for democracies of recent origin, where the election procedure itself is a new phenomenon, and the observed phenomena and their persistence or variability has a short tradition. This is the situation of Poland, which took the road to democracy in 1989 after the post-war communist period. It was made possible by two waves of protests by the Polish Solidarity movement in 1980–1981 and 1988–1989, the launch of ‘perestroika’ by Mikhail Gorbachev in 1985 and the 1989 ‘Round Table’ agreement between the Polish democratic opposition and the Polish communist authorities. That same year, partially democratic elections were held, resulting in Poland’s first non-communist government since World War II^13,14^. This made it possible to carry out a change in the political system. In 1990, the first fully free presidential elections were held, followed by the first fully free parliamentary elections in 1991^15^. Since then, despite various political tensions, the system of democratic elections in Poland has functioned without major problems^16^. This is a good circumstance for studying all the issues mentioned above. For this to be possible, it is necessary to have a GIS dataset that allows easy and yet effective substantive analysis.

In our experience, the best reference point for electoral analysis in Poland is municipalities, counties and electoral districts (constituencies), although each of these units has its own advantages and disadvantages. At first glance, municipalities may appear to be the best administrative unit for various types of analysis and comparison. First, they are the lowest level of Poland’s administrative division, which at the same time is the basis for the functioning of local government with broad powers of public authority. The country’s nearly 2,500 municipal-level units allow one to learn in detail about the country’s spatial differentiation in various spheres, including voting behaviour. However, the division into municipalities is not always optimal for scientific research.

First of all, units of this size are strongly differentiated in terms of area and population, which makes it difficult to understand and properly assess, appearing on the map, spatial differentiation. The municipality-city of Warsaw currently has more than 1,400,000 eligible voters, while many peripheral rural municipalities have less than 2,000. Second, much data of a socio-economic nature that could be useful for comparative analysis within the framework of so-called electoral ecology is not available in Polish datasets at the municipality level, but at a higher level of aggregation, such as counties.

The basic units for electoral analysis should be electoral districts. It is in them that the battle for parliamentary seats takes place both between parties and within party lists. The results in the districts thus determine the party and personal composition of parliament. Analysis of the variability of voting behavior and the characteristics of local society, can have important implications for building the electoral strategies of individual political parties. On the other hand, there are only 41 electoral districts for the lower house of parliament in Poland, which means that the variability shown at this scale obscures many of the spatial differences evident at the local scale. There is also no direct data for them on sundry socio-economic variables. However, they can be aggregated from smaller units, although data averaged over large units of data are less useful in the search for cause-and-effect relationships. In this context, a rather convenient unit of analysis is the districts in the elections to the Senate (the upper house of parliament), of which there are 100 (as many as there are seats in the Senate). In addition, they mostly have a similar area and, by definition, a similar population (each senator must represent a similar number of voters). For this reason, in our dataset we propose to aggregate electoral data to these two territorial units: Sejm districts and Senate districts.

The lack of a widely available platform for electoral-spatial analysis was at the heart of the Center for Electoral Analysis’ research project, “Electoral Geoportal”, funded by sources from the Polish Ministry of Education and Science^17^. The purpose of the Electoral Geoportal is to present the results of Polish general elections in electronic and interactive form. The development of digital techniques, including cartographic visualization, allows for many opportunities to expand analysis in this area. Of particular importance is the prospect of individual users creating their own cartographic and graphic summaries. Through the website, this becomes possible for a wide audience.

The electoral geoportal was inspired by the Electoral Atlas of Poland, published in 2018 by the Institute of Geography and Spatial Planning of the Polish Academy of Sciences in Warsaw also in traditional, i.e. paper form^18^ and placed in PDF format in the Digital Repository of Scientific Institutes^19^. In 2023, its second revised and expanded edition was published, the publisher of which, alongside IGiPZ PAN, was the Sejm Publishing House (an institution associated with the Polish parliament)^20^. This atlas contains, among other things, a collection of more than 700 maps, presenting the results and spatial analysis of general elections in Poland, mainly from the period 1989–2015/2020. The Electoral Atlas of Poland project was carried out at IGiPZ PAN since 2011 and funded by the National Science Center^21^. The knowledge, experience and data gathered by the editors and authors became the starting point for a new scientific and expert idea called the Electoral Geoportal, implemented under the auspices of the Jagiellonian Academy in Torun (https://www.geoportalwyborczy.pl/en-gb/about)^22^.

As a result of the work on the Electoral Atlas of Poland and then the Electoral Geoportal, comprehensive and structured datasets of general elections held in Poland between 1990 and 2024 have been created. They include data from different types of elections and in different territorial arrangements, appropriate for a given electoral period (year). We felt that it was worth the extra effort and an attempt to unify these datasets to ensure comparability in time and space, and thus make it possible to advance research in which these variables are crucial in explaining social phenomena and processes.

It seems to us that the dataset being made available is the first such extensive and comprehensive set of electoral data for Central and Eastern Europe, and certainly for Poland. We call the dataset we have developed GeoElections Poland 1.0, which suggests its future development. We plan the following changes and improvements:

- updating the voting data with subsequent general elections;
- providing aggregated and comparable data at lower and lower levels of spatial aggregation (the smallest unit is the electoral district, but data at such a territorial level, due to frequent changes in the boundaries of these units, would not be possible to compare over time);

Our work was carried out under publicly funded grants. Therefore, we believe that access to the results of our work should be open, so that anyone can use them for information or use them for their own analysis and studies.

## Methods

The work that led to the creation of the datasets consisted of several stages and courses of action. First, in view of the diversity of the electoral system, a selection of elections for development was needed. Second, it was needed to develop spatial and subject aggregation rules related to the specifics of the elections. Thirdly, there was a need to choose the optimal method of data sharing, providing opportunities for data analysis and presentation.

### Selection of general elections

The electoral system in Poland allows voting in general elections such as for Parliament (Sejm and Senate), for the President of the Republic of Poland (maximum two rounds of voting), for the European Parliament, in local elections (provincial assemblies, elections for county councils, municipalities, for heads of municipalities, mayors and city presidents), as well as voting in referendums. In total, between 1990 and 2024, Poland held about 40 votes covering the entire country, in which a total of about 2 million candidates took part passively (about 1% of the country’s population, since very often the same person ran multiple times in different elections and with different results). Elections between 1990 and 2024 were held in some 20,000–30,000 voting districts (local voting commissions), changing territorial boundaries over time. These numbers indicate the many challenges faced in creating a unified and comparable source base that can meet the rigors of application in scientific analysis. In this, it has become impossible to develop a complete base, including all the results of support and voting results in all elections in all voting districts.

It was decided that the key premise, determining the selection of elections for the dataset, is their scientific usefulness in explaining the phenomena and processes of electoral geography. Thus, the base is created first based on elections with the greatest significance and social resonance. These are in the period 1990–2024:

- 10 parliamentary elections to the Sejm (1991, 1993, 1997, 2001, 2005, 2007, 2011, 2015, 2019, 2023);
- 7 presidential elections (1990, 1995, 2000, 2005, 2010, 2015, 2020), including 6 first- and second-round elections (two different votes two weeks apart), and in one election (2000) there was only one round due to the election of a candidate already in the first round.
- 5 elections to the European Parliament (2004, 2009, 2014, 2019, 2024).

In addition, between 1990 and 2024, there were 9 nationwide local elections (from 1990–2018 every 4 years, from 2024 for a 5-year term) and 6 referendums (usually on different dates than other general elections). We do not deal with them due to either a different type of voting, generally difficult to compare with turnout and support in general elections (referendums), or due to the particularly high diversity of local actors (in the case of local elections).

The full list of elections gated from consideration is presented in Table 1. In the dataset of parliamentary elections, only the results of voting for the lower house of parliament (Sejm) are included. This restriction was made for two reasons. First, the upper house was omitted because the voting results have much less analytical value due to the much greater individuality of the vote and the rather complex and changing voting system over time, which at different times was essentially quasi-one-mandate. This makes it impossible to make meaningful comparisons with the parties taking part in voting for, say, the parliament (Sejm). Second, the lower house of parliament has much greater prerogatives of power, which translates into interest in this form of elections and voting preferences. Third, the technical factor is important, since even if these results could be compiled in some comparable way, the base created would be many times larger than for other general elections (for some elections it would have to include up to several hundred individual candidates each time).

**Table 1:** Elections prepared and made available in the dataset GeoElections Poland 1.0.

Date of elections	Type of elections	Separate voting committees (submitted voting lists)
November 25, 1990	Presidential (1st round)	6
December 9, 1990	Presidential (2nd round)	2
October 27, 1991	Parliamentary (Sejm)	111
September 19, 1993	Parliamentary (Sejm)	35
November 5, 1995	Presidential (1st round)	13
November 19, 1995	Presidential (2nd round)	2
September 21, 1997	Parliamentary (Sejm)	20
October 8, 2000	Presidential (1st round)	12
September 23, 2001	Parliamentary (Sejm)	14
June 13, 2004	European Parliament	21
October 9, 2005	Presidential (1st round)	12
October 23, 2005	Presidential (2nd round)	2
September 25, 2005	Parliamentary (Sejm)	22
October 21, 2007	Parliamentary (Sejm)	10
June 7, 2009	European Parliament	12
June 20, 2010	Presidential (1st round)	10
July 4, 2010 2010	Presidential (2nd round)	2
October 9, 2011	Parliamentary (Sejm)	11
May 25, 2014	European Parliament	12
May 10, 2015	Presidential (1st round)	11
May 24, 2015	Presidential (2nd round)	2
October 25, 2015	Parliamentary (Sejm)	17
May 26, 2019	European Parliament	9
October 13, 2019	Parliamentary (Sejm)	10
June 28, 2020	Presidential (1st round)	11
July 12, 2020	Presidential (2nd round)	2
October 15, 2023	Parliamentary (Sejm)	12
June 9, 2024	European Parliament	11

Source: based on National Electoral Commission (Państwowa Komisja Wyborcza), National Election Office (Krajowe Biuro Wyborcze).

### Gathering and organizing data on turnout and support results

Access to electoral data in Poland, comparable at different levels of territorial aggregation, is very complicated, despite the law on access to public information, which complies with European Union standards. The institution responsible for the organization of elections, their conduct, the counting of votes and the announcement of election results is the State Election Commission (PKW). Its website provides the results of parliamentary elections from 2001–2023 and the results of presidential elections from 2000–2020 (https://pkw.gov.pl/wybory-i-referenda)^23^.This data is publicly available, as stipulated in Article 163 of the Electoral Code in force in Poland (https://isap.sejm.gov.pl/isap.nsf/DocDetails.xsp?id=wdu20110210112), which obliges the State Electoral Commission to publish statistical studies containing detailed information on the results of voting and elections, and to make the results of voting and elections available in the form of an electronic document^24^. In addition to infographics with cartographic elements showing election results at the level of the country, provinces, electoral districts (constituencies), counties, municipalities and voting districts (local voting commissions), there are also Excel and CSV files with election results at various levels of territorial division.

Earlier presidential and parliamentary elections from 1990–1997, but without visualization of the results, have been available since around 2020 on the website of the National Election Office (https://danewyborcze.kbw.gov.pl)^25^, the institution that provides administrative and technical services for the PKW. There are electoral data presented at different levels of the country’s administrative division (constituencies, counties, municipalities, voting districts). This information is also publicly available, in accordance with the aforementioned Article 163 of the Electoral Code^24^. Unfortunately, there are no infographics to bring citizens and the public closer to the knowledge of the spatial aspects of all these elections.

Election data in Poland, according to the electoral law, contains a number of variables, allowing detailed insight not only into the results, but also into many details of the organization of the elections (e.g., in terms of postal voting, invalid votes, etc.). For the purposes of the dataset provided, the following election data were selected: the number of eligible voters, the number of votes cast (turnout), the number of valid votes, the number of valid votes cast for a given list (committee). These data were checked for accuracy (e.g., vote totals) and territorial coverage, and then processed into uniform municipal datasets and catalogued. The collected and catalogued (described) dataset is probably the best documented and archived set of electoral data by municipality in Poland.

We have also supplemented our dataset with a classification of election participants by type, group, etc., based on their ideological and political character. This will undoubtedly improve the comparability of election data, given the frequent changes in the names of political entities in Poland and their short period of operation, while maintaining a clear division of the political scene according to criteria that are understandable to the public. For this reason, we have adopted a political division into four options that is well established in Polish debate: left-wing, right-wing, liberal, people’s (rural-agrarian) and local (national and ethnic) minorities^3^. Throughout the period 1990–2024, the largest number of parties and groups were right-wing (133), followed by liberal (102).

### Territorial aggregation unit

During the implementation of the Electoral Atlas of Poland project, vector maps were made of the boundaries of municipalities in each election year. The municipalities were assigned to their corresponding counties and provinces (in the new administrative-territorial division since 1999), as well as to each electoral districts (constituencies). Their number in the 1991–2023 elections to the Sejm varied from 37 to 52. For this purpose, especially for the years before about 2010, when there were no widely available geodetic datasets of administrative boundaries, the existing base material collected at the IGiPZ PAN (a research institution specializing in the cartographic development of the Polish territory for years) was used. In this, it was necessary to take into account the rather numerous changes in administrative boundaries of municipalities, especially in 1990–1995.

Election ‘raw’ data were aggregated first to the level of municipalities in a given year, and then to the corresponding electoral districts (constituencies) in a given year. In the end, some 20 sets of maps and accompanying data were produced, corresponding to those election years. These were not comparable to each other at more distant time intervals due to changes in the number of municipalities and their boundaries.

**Fig. 1:**
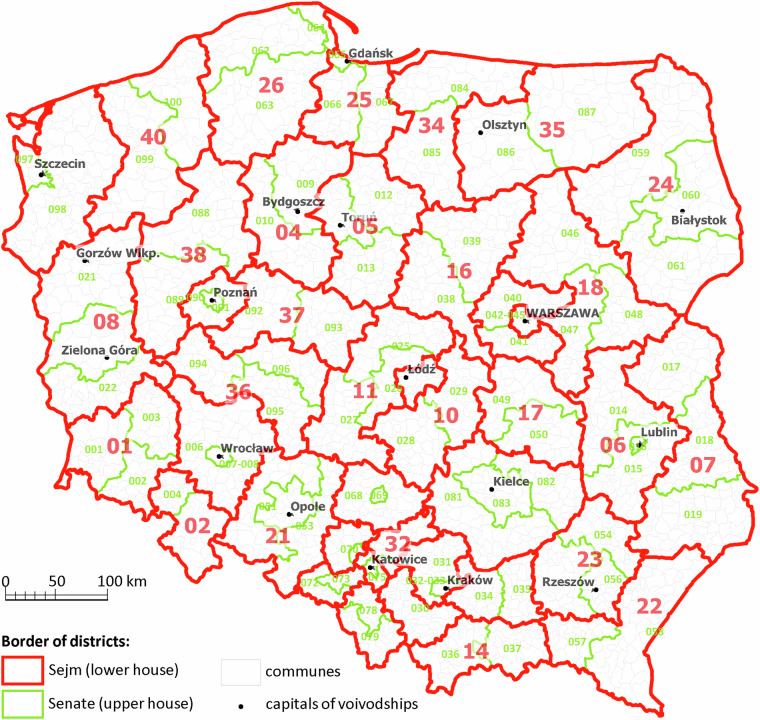
Map of the Sejm and Senate electoral districts where election data is provided. Numeration: Electoral districts of Sejm: 01 - Legnica; 02 - Wałbrzych; 03 - Wrocław; 04 - Bydgoszcz; 05 - Toruń; 06 - Lublin; 07 - Chełm; 08 - Zielona Góra; 09 - Łódź; 10 - Piotrków Trybunalski; 11 - Sieradz; 12 - Kraków; 13 - Kraków; 14 - Nowy Sącz; 15 - Tarnów; 16 - Płock; 17 - Radom; 18 - Siedlce; 19 - Warszawa; 20 - Warszawa; 21 - Opole; 22 - Krosno; 23 - Rzeszów; 24 - Białystok; 25 - Gdańsk; 26 - Słupsk; 27 - Bielsko-Biała; 28 - Częstochowa; 29 - Katowice; 30 - Bielsko-Biała; 31 - Katowice; 32 - Katowice; 33 - Kielce; 34 - Elbląg; 35 - Olsztyn; 36 - Kalisz; 37 - Konin; 38 - Piła; 39 - Poznań; 40 - Koszalin; 41 - Szczecin. Electoral districts of Senate: 001 - Legnica; 002 - Legnica; 003 - Legnica; 004 - Wałbrzych; 005 - Wałbrzych; 006 - Wrocław; 007-008 - Wrocław; 009 - Bydgoszcz; 010 - Bydgoszcz; 011 - Toruń; 012 - Toruń; 013 - Toruń; 014 - Lublin; 015 - Lublin; 016 - Lublin; 017 - Chełm; 018 - Chełm; 019 - Chełm; 020 - Zielona Góra; 021 - Zielona Góra; 022 - Zielona Góra; 023-024 - Łódź; 025 - Sieradz; 026 - Sieradz; 027 - Sieradz; 028 - Piotrków Trybunalski; 029 - Piotrków Trybunalski; 030 - Kraków; 031 - Kraków; 032-033 - Kraków; 034 - Tarnów; 035 - Tarnów; 036 - Nowy Sącz; 037 - Nowy Sącz; 038 - Płock; 039 - Płock; 040 - Warszawa; 041 - Warszawa; 042-045 - Warszawa; 046 - Siedlce; 047 - Siedlce; 048 - Siedlce; 049 - Radom; 050 - Radom; 051 - Opole; 052 - Opole; 053 - Opole; 054 - Rzeszów; 055 - Rzeszów; 056 - Rzeszów; 057 - Krosno; 058 - Krosno; 059 - Białystok; 060 - Białystok; 061 - Białystok; 062 - Słupsk; 063 - Słupsk; 064 - Słupsk; 065 - Gdańsk; 066 - Gdańsk; 067 - Gdańsk; 068 - Częstochowa; 069 - Częstochowa; 070 - Katowice; 071 - Katowice; 072 - Bielsko-Biała; 073 - Bielsko-Biała; 074 - Katowice; 075 - Katowice; 076 - Katowice; 077 - Katowice; 078 - Bielsko-Biała; 079 - Bielsko-Biała; 080 - Katowice; 081 - Kielce; 082 - Kielce; 083 - Kielce; 084 - Elbląg; 085 - Elbląg; 086 - Olsztyn; 087 - Olsztyn; 088 - Piła; 089 - Piła; 090 - Poznań; 091 - Poznań; 092 - Konin; 093 - Konin; 094 - Kalisz; 095 - Kalisz; 096 - Kalisz; 097 - Szczecin; 098 - Szczecin; 099 - Koszalin; 100 - Koszalin. Source: own elaboration based on National Electoral Commission (Państwowa Komisja Wyborcza).

The final and most difficult task was the development of an aggregate, synthetic and unified dataset that would allow comparisons across any cross-section of time for the same territorial units, and which is provided in this study. Therefore, for comparative purposes in this study, it was necessary to propose a single and uniform territorial aggregation. It was decided to use the division into 41 constituencies for the Sejm and 100 constituencies for the Senate, which has been in effect since 2011 (Fig. 1). At the same time, in order to adjust the data to the division into cities and municipalities in Poland, minor adjustments were needed, resulting from the division of constituencies in large cities, i.e. the aggregation of two constituencies in Krakow, Lodz and Wroclaw, and five constituencies in Warsaw. Thus, the total number of senatorial constituencies was reduced from 100 to 94. This allows reliable comparisons of voting behaviour in units that are clearly spatially differentiated (regionally), but fairly homogeneous in terms of administrative and settlement status (senatorial constituencies are especially self-contained large cities and their suburban zones, or so-called functional urban areas).

For those who, for whatever reason, need more detailed data at the municipal level, we recommend using the aforementioned repositories of the National Electoral Commission^23^ and the National Electoral Office^25^. They contain raw electoral data on municipalities (and even smaller units) in Excel format. This data can be linked to vector maps, which can be downloaded from the Polish public portal Geoportal (https://www.geoportal.gov.pl/pl/dane/panstwowy-rejestr-granic-prg/). These maps are only available for the years 2005-2025^26^, but after some adjustments, they can also be used for earlier elections. Unfortunately, data organised in this way is not suitable for precise comparisons, due to the changing number of municipalities over time (units were merged or divided) and changes in the boundaries between them.

### Organization of the dataset

The dataset is provided as a relational dataset in XLS file. The sheet “GeoElections Poland 1.1 data” contains data organized by characteristics, absolute values (number of eligible voters, number of votes cast - turnout, number of valid votes, number of valid votes cast for a given list/committee, political option). All presidential candidates and parliamentary electoral lists were given unified and easily identifiable abbreviations in the dataset, and an explanation of these abbreviations was included in the next sheet (‘Commentary’). A pivot table was generated from the GeoElections Poland 1.1 date sheet (byElectoralDistrict sheet, in which data for the Sejm and Senate districts are summarized in multidimensional tables as separate columns (nearly 500 columns with data for each year, election, committee).

The main dataset (sheet “GeoElections Poland 1.0 data”) contains 12 columns:

- Year – election year (1990–2024),
- ElectionType – type of election (Presidential 1st round, Presidential 2nd round, Sejm/lower house of parliament, European Parliament),
- AbbreviationInSheet – abbreviation in sheet, For example, the abbreviation E2024PIS refers to the 2024 European Parliament elections, votes cast for Law and Justice,
- OriginalName – official name in Polish of the political party or presidential candidate (first and last name),
- Option – one of five assigned political options (left-wing, right-wing, liberal, people’s/rural-agrarian and local/national and ethnic) minorities),
- Votes – the number of votes cast for a party list or candidate, as well as the number of eligible voters and voter turnout,
- NumberSejm41 – district number in Sejm elections, standardized for the entire period 1990–2024 to 41 districts,
- NameSejm41 – as above, with the name of the district,
- NumberNameSejm41 – combination of the number and name of the Sejm district,
- NumberSenat94 – district number in Senate elections, standardized for the entire period 1990–2024 to 94 districts,
- NameSenat94 – as above, with the name of the largest urban center (Senate election districts do not have their own names, only numbers along with the seat of the district electoral commission),
- NumberNameSenate94 – combination of the number and name of the Senate district.

The vector map (underlay) dataset of parliamentary and senatorial districts was prepared in two SHP files in EPSG 4326 (WGS84) mapping. The district (constituencies) boundaries are generalized to a scale of 1:1,000,000. Each district has an individual code and name, completely compatible with the codes and names in the XLS file with election data. It is also possible to generate a map with more detailed boundaries, it is possible to download data on the boundaries of municipalities from the State Register of Borders (https://dane.gov.pl/pl/dataset/726,panstwowy-rejestr-granic-i-powierzchni-jednostek-podziaow-terytorialnych-kraju)^27^, in which case it is necessary to download district codes from the Communes sheet, assigned there to individual municipalities (each municipality according to the 2024 division was assigned to the corresponding parliamentary or senatorial district).

## Data Records

We placed our dataset in the figshare (10.6084/m9.figshare.28869773)^28^. The set consists of files in SHP format for two levels of territorial aggregation (constituencies for the Parliament and the Senate). The name of the unit and its code are added to the vector map, allowing data to be linked to an Excel dataset with turnout and unified voting results for all 22 general elections from 1990–2024 (Sejm, President, Europarliament).

## Technical Validation

The main problem we encountered in the development of our datasets was changes to territorial unit codes, changes to unit boundaries, and the splitting and merging of units as a result of decisions by state administrative authorities. We had to detect and account for these changes in order to keep the results up-to-date and comparable. This procedure was particularly important for the period before about 2000, when a different territorial and electoral division was in force. The complicated procedure of connecting and merging data and converting units could have resulted in numerical errors. For this reason, data validation was needed. Therefore, we checked all datasets for consistency in the number of votes. At each level of aggregation, we added up the number of votes for each grouping and checked that the numbers corresponded to those at a higher level of aggregation provided by the State Electoral Commission (municipalities > Senate districts > Sejm districts > country). Nowhere did we find any difference.

## Data Availability

Data concerning elections in Excel and SHP files in selected territorial units are available in the figshare (10.6084/m9.figshare.28869773)^28^

## Code availability

We used no customized software for our datasets^28^.
